# Supplementary material for: Persistent activity in human parietal cortex mediates perceptual choice repetition bias
Source: Nat Commun. 2022 Oct 12;13:6015. doi: 10.1038/s41467-022-33237-5 (PMC9556658; doi:10.1038/s41467-022-33237-5)
Supplement: Supplementary file 1 — Supplementary Information [file 41467_2022_33237_MOESM1_ESM.pdf]

Supplementary Figures

Persistent Activity in Human Parietal Cortex Mediates Perceptual Choice Repetition Bias

Anne E. Urai & Tobias H. Donner

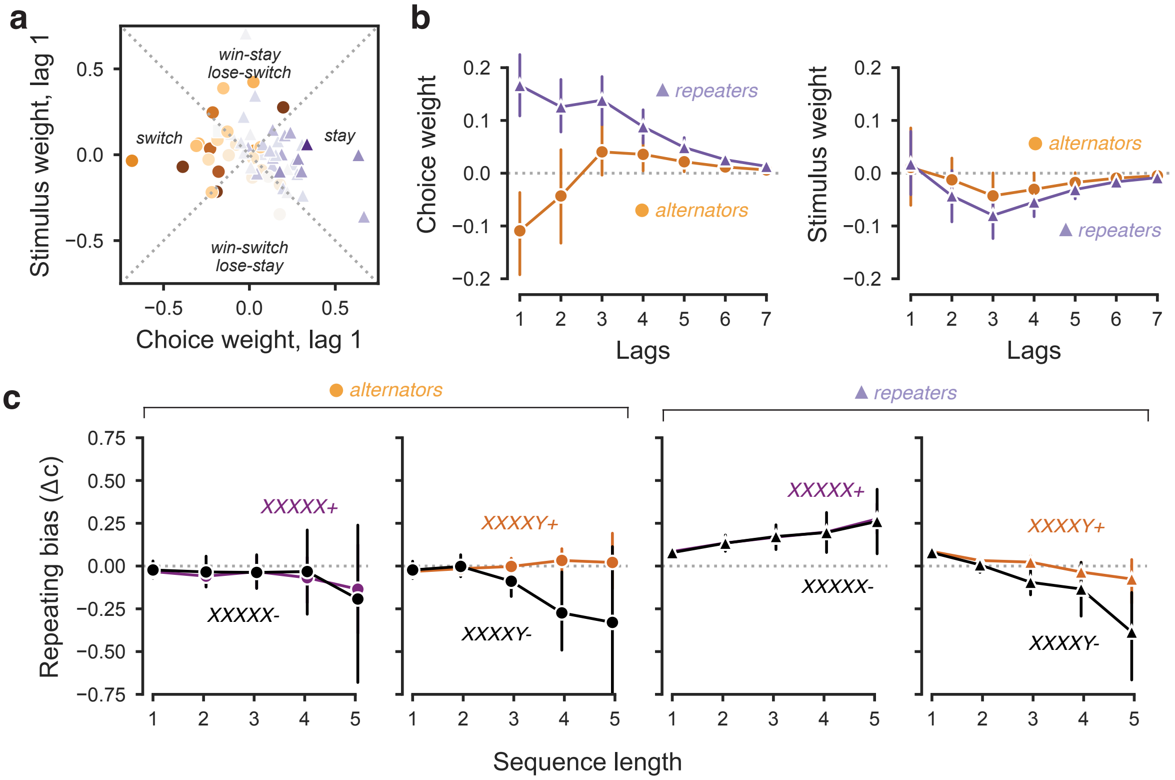

Figure S1. Multi-trial behavioral choice history biases. *(a) Choice weights plotted against their corresponding stimulus weights show each individual's decision strategy [1]. The left and right quadrants indicate purely choice-based strategies ('switch' vs. 'stay'), whereas the top and bottom quadrants indicate outcome-dependent choice strategies ('win-stay/lose-switch' vs. 'win-switch/lose-stay'). Colors indicate individual choice repetition probability, with circles for alternators and triangles for repeaters.* ***(b)*** *Choice and stimulus weights, averaged within subgroups of alternators (n = 25) and repeaters (n = 34), for lags 1-7.* ***(c)*** *Multi-trial repeating sequences ending in a repetition (XX) or alternation (XY), separately for alternators (left, n = 25) and repeaters (right, n = 34). Unlike behavioral sequences in rats [2], these do not consistently differ based on the outcome (correct +, colored markers vs. error -, black markers) of the last trial. Data are shown as mean +- 95% bootstrapped confidence intervals.*

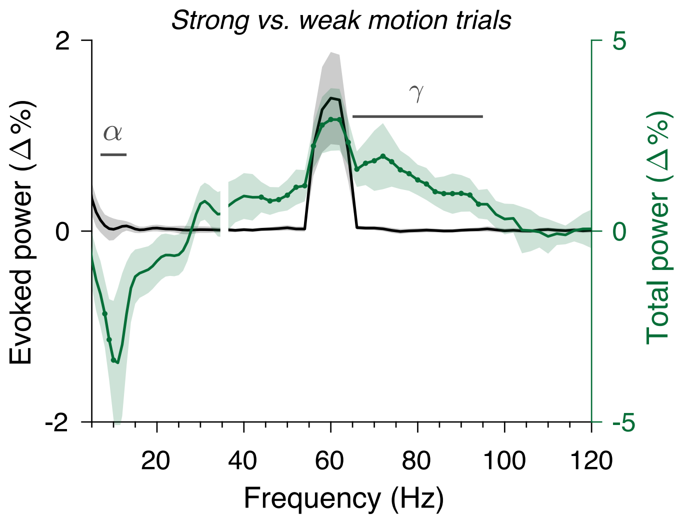

Figure S2. Evoked and total visual responses. *Power spectra of the difference between strong and weak visual motion (250-750 ms after test stimulus onset, expressed in % from the average pre-reference baseline). To obtain these sensor-level spectra, we used the occipital electrodes from a split- half sensor definition shown in Figure 2b. Power was either computed after averaging trials in the time domain (evoked power, grey) or computed on single trials before averaging (total power, green). Green dots indicate frequencies that are significantly different for the contrast between strong and weak visual motion, defined as participating in > 80% of the timepoints). These spectra show that the two coherent motion stimuli elicited a response around the screen refresh rate (60 Hz +/- spectral smoothing box, see Methods), which was phase-locked to stimulus onset and hence dissociable from the high-frequency response occurring at random phase (Figure 2a). Data are shown as mean +- 95% bootstrapped confidence intervals (n = 60).*

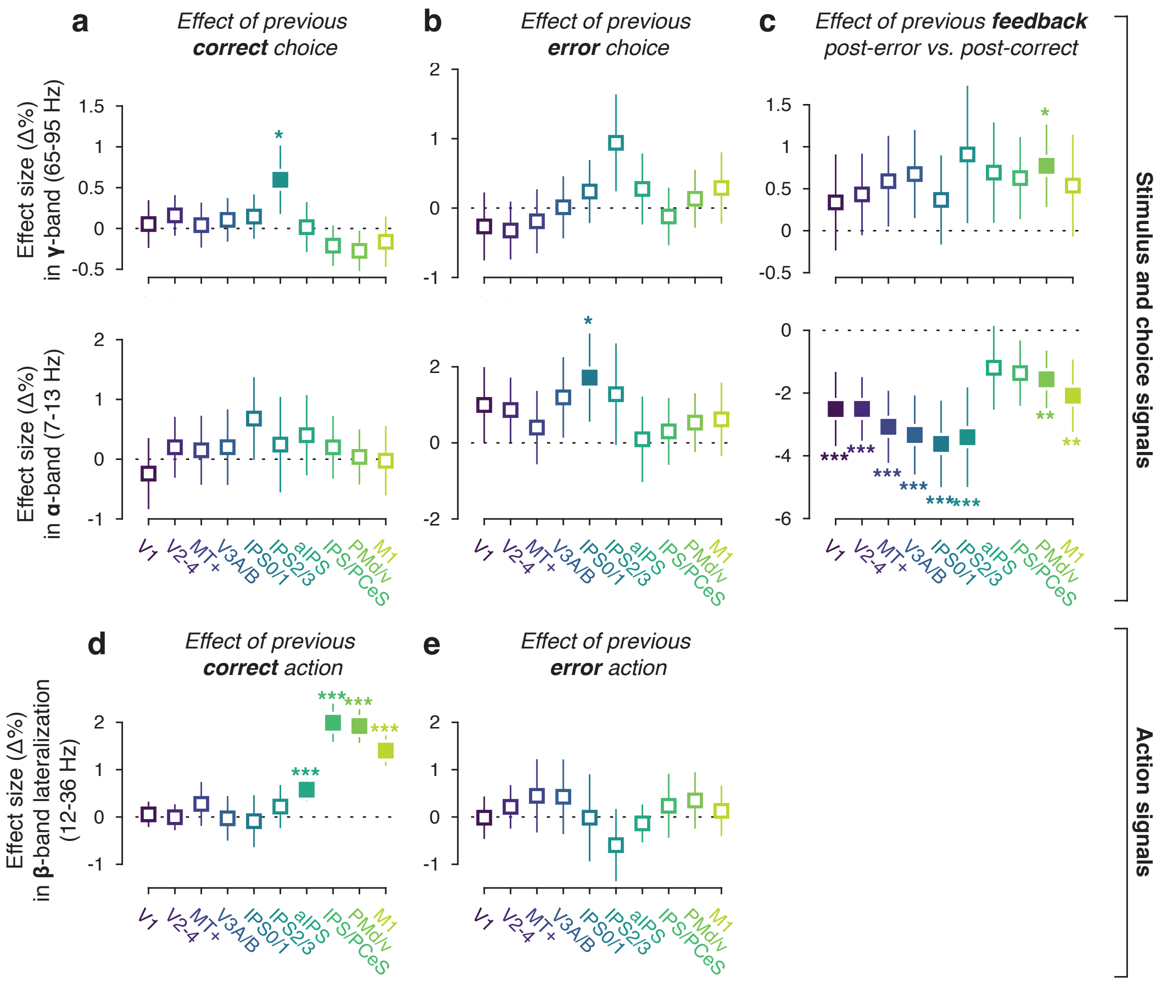

Figure S3. Outcome-dependent history signals. *As Figure 4 (effect sizes from a general linear mixed model, see Methods), but with separate predictors for previous correct **(a)** and error **(b)** choices, and correct **(d)** and error **(e)** motor actions (all n = 60). **(c)** The main effect of previous error vs. correct outcomes. Data are shown as mean +- 95% confidence intervals.*

In a model with interaction terms between previous choices and previous outcomes, this interaction did not reach significance in either IPS2/3 gamma: (main effect of previous choice = 0.6780, CI [0.2578, 1.0981, p = 0.0016; main effect of previous feedback = 0.9085, CI [0.0900, 1.7270], p = 0.0296; interaction = 0.2384, CI [-0.5801, 1.0570], p = 0.5681] or IPS0/1 alpha (main effect of previous choice = 0.7539, CI [0.0543, 1.4534], p = 0.0347; main effect of previous feedback = -3.6227, CI [-4.9997, -2.2456], p < 0.0001; interaction = 0.9189, CI [-0.4435, 2.2814], p = 0.1862).

The global, error-driven component of posterior alpha-power modulations has been observed in previous EEG work[3]*. Critically, the specific choice history signal in IPS0/1 alpha was still present after removing the global component (computed across visual field maps except IPS0/1) via linear regression (Methods): main effect of previous choice on the residual IPS0/1 alpha signal of 0.0096 (CI [0.0040, 0.0152], p = 0.0007).*

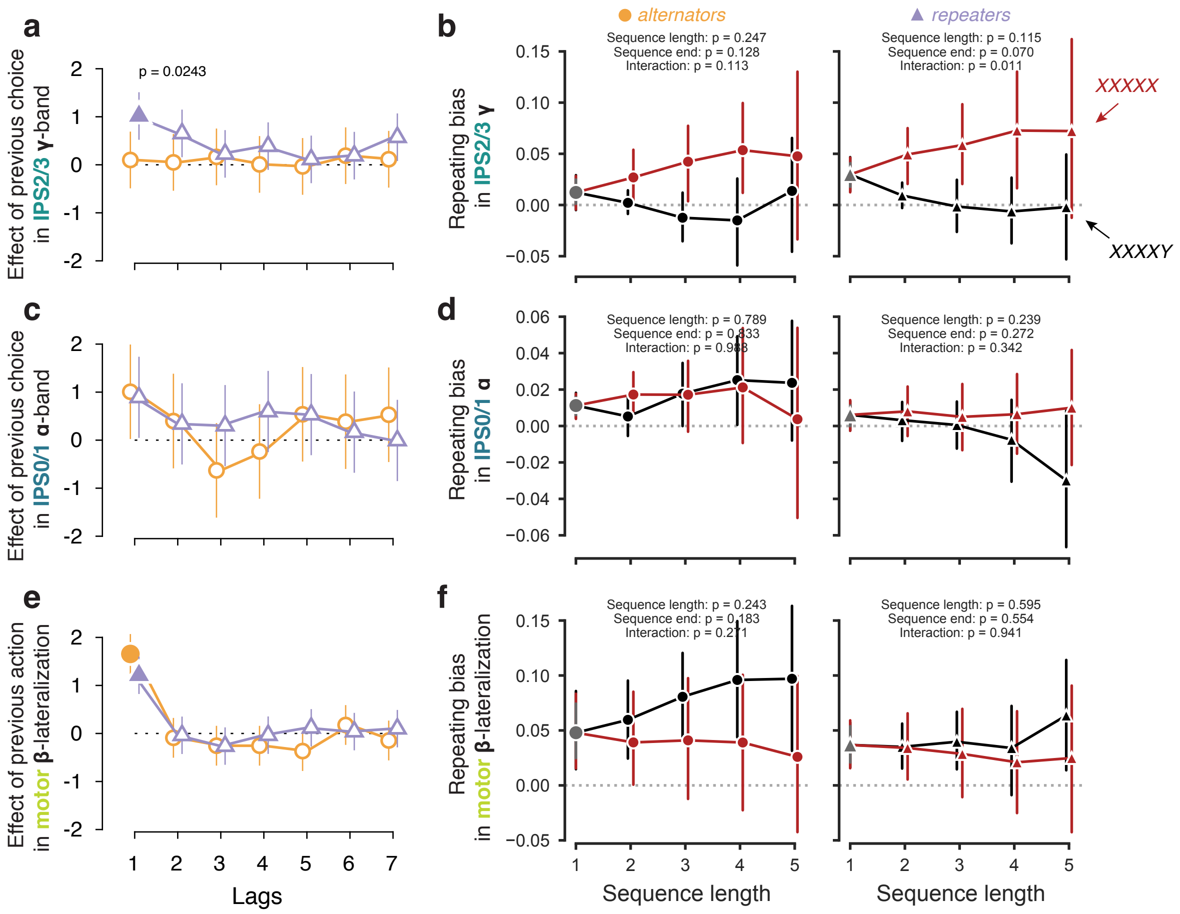

Figure S4. Multi-trial effects in neural history signals. (a, c, e) *As Figure 4d-h but for regression weights of previous choices or actions up to 7 past trials, separately for alternators (n = 25) and repeaters (n = 34).* ***(b, d, f)*** *Build-up of neural history signals across multi-trial streaks. As Figure 1 but for the neural repeating bias, i.e. the average tendency of each neural signal to reflect the previous choice. Data are shown as mean +- 95% confidence intervals.*

*Although the interaction between sequence length and sequence end is significant for IPS2/3 gamma in repeaters only (**b**), a three-way ANOVA with subgroup as a factor did not show a significant interaction with subgroup F(4) = 0.401, p = 0.808.*

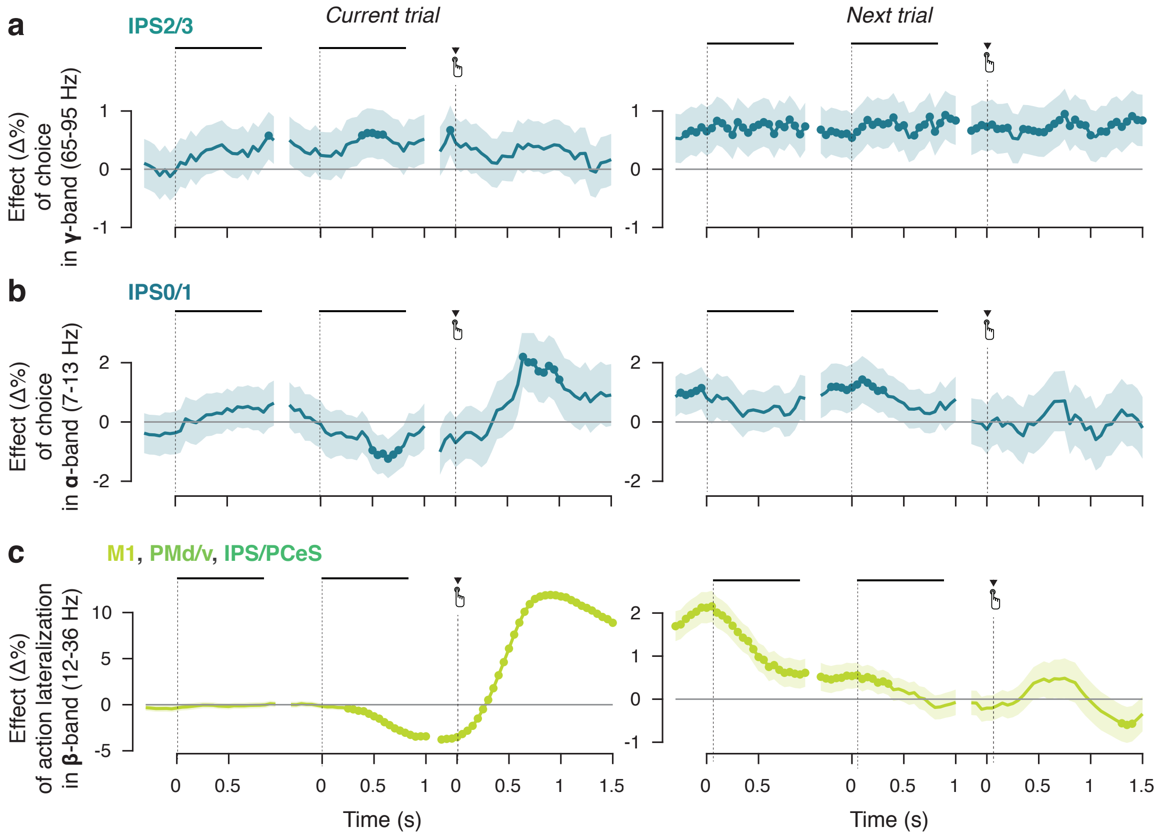

Figure S5. Within-trial time course of choice history effects. *As Figure 5, but across all observers (n = 60) for **(a)** IPS2/3 gamma, **(b)** IPS0/1 alpha, **(c)** motor (pooled M1, PMd/v, IPS/PCeS) beta. Data are shown as mean +- 95% confidence intervals.*

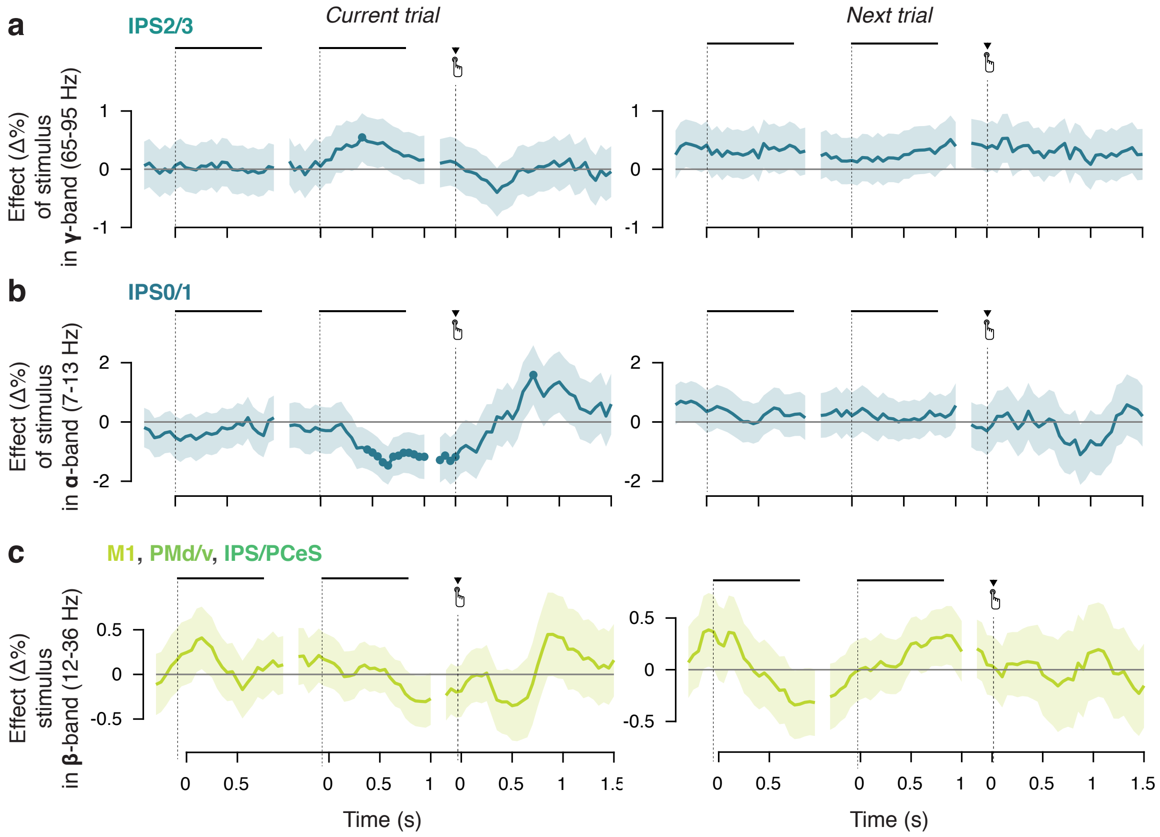

Figure S6. Within-trial time course of stimulus history effects. *As Figure 5, but for current and previous stimuli (rather than choices or actions) across all observers (n = 60) for* ***(a)*** *IPS2/3 gamma,* ***(b)*** *IPS0/1 alpha,* ***(c)*** *motor (pooled M1, PMd/v, IPS/PCeS) beta. Data are shown as mean +- 95% confidence intervals.*

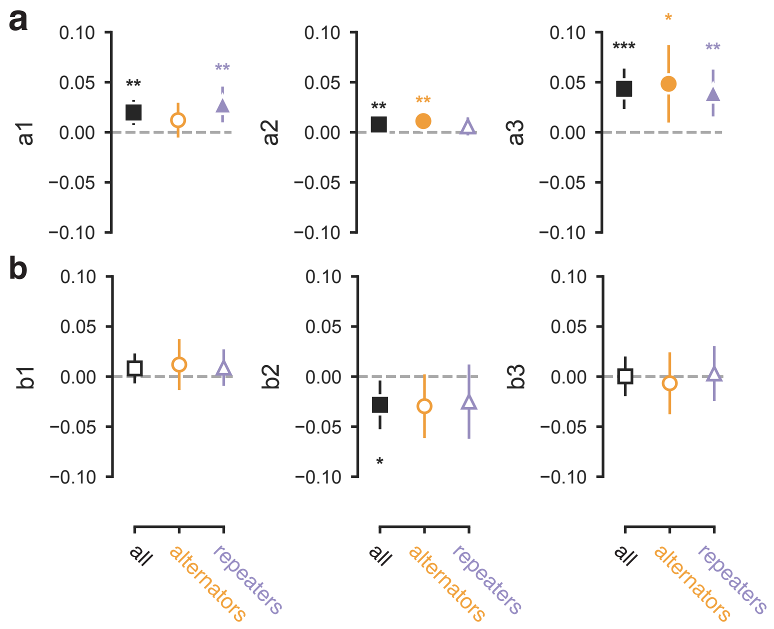

Figure S7. Mediation paths. *As Figure 6, but showing the* ***(a)*** *a-path and* ***(b)*** *b-path separately. Parameter estimates for the complete group (n = 60) as well as both subgroups (n = 25 alternators in orange, n = 34 repeaters in purple). Data are shown as mean +- 95% confidence intervals. Unpaired t-tests between groups did not show significant differences on these individual paths.*

Effect sizes for all participants: ***a1*** *t(59) = 3.166, p = 0.0024;* ***a2*** *t(59) = 2.675, p = 0.0097;* ***a3*** *t(59) = 4.305, p = 0.0001;* ***b1*** *t(59) = 1.091, p = 0.2796* ***b2*** *t(59) = -2.325, p = 0.0235,* ***b3*** *t(59) = 0.026, p = 0.9792. Alternators:* ***a1*** *t(24) = 1.442, p = 0.1624;* ***a2*** *t(24) = 3.094, p = 0.0050;* ***a3*** *t(24) = 2.583, p = 0.0163;* ***b1*** *t(24) = 0.971, p = 0.3412;* ***b2*** *t(24) = -1.917, p = 0.0672;* ***b3*** *t(24) = -0.445, p = 0.6605. Repeaters:* ***a1*** *t(33) = 3.175, p = 0.0032;* ***a2*** *t(33) = 1.334, p = 0.1914;* ***a3*** *t(33) = 3.421, p = 0.0017;* ***b1*** *t(33) = 1.000, p = 0.3245; b2 t(33) = -1.374, p = 0.1788;* ***b3*** *t(33) = 0.226, p = 0.8224.*

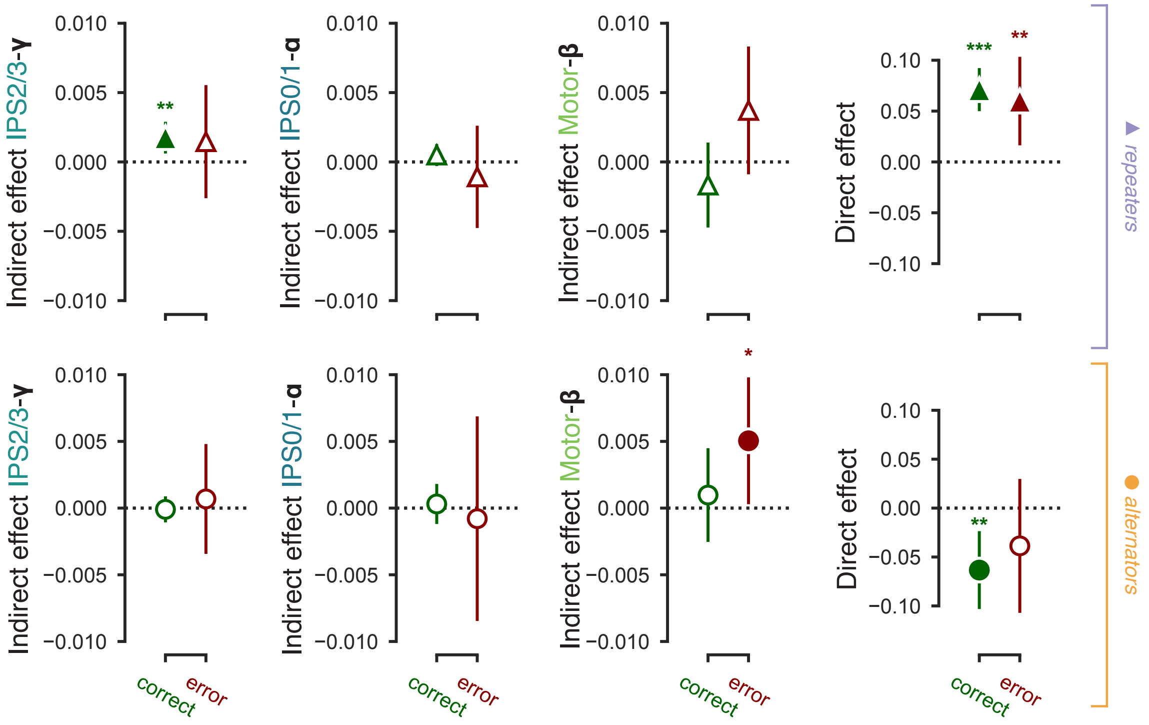

Figure S8. Mediation results after correct and error trials. *As Figure 6, but splitting trials by the feedback of the previous trial. * 0.01 < p < 0.; *** p < 0.001; filled markers, p < 0.05.*

Parameter estimates for repeaters (n = 34, top): IPS2/3-gamma correct t(33) = 2.205, p = 0.0345; error t(33) = 0.347, p = 0.7308; IPS0/1-alpha correct t(33) = 1.300, p = 0.2025; error t(33) = 0.509, p = 0.6139; Motor-beta correct t(33) = -0.560, p = 0.5789; error t(33) = 1.430, p = 0.1621; direct path correct t(33) = 5.686, p < 0.0001; error t(33) = 3.451, p = 0.0015. Parameter estimates for alternators (n = 25, bottom): IPS2/3-gamma correct t(24) = 1.169, p = 0.2539; error t(24) = 0.802, p = 0.4302; IPS0/1-alpha correct t(24) = 0.216, p = 0.8307; error t(24) = -1.782, p = 0.0875; Motor-beta correct : t(24) = -0.488, p = 0.6301; error t(24) = 2.261, p = 0.0331; direct path correct t(24) = -1.503, p = 0.1459; error t(24) = -1.106, p = 0.2796.

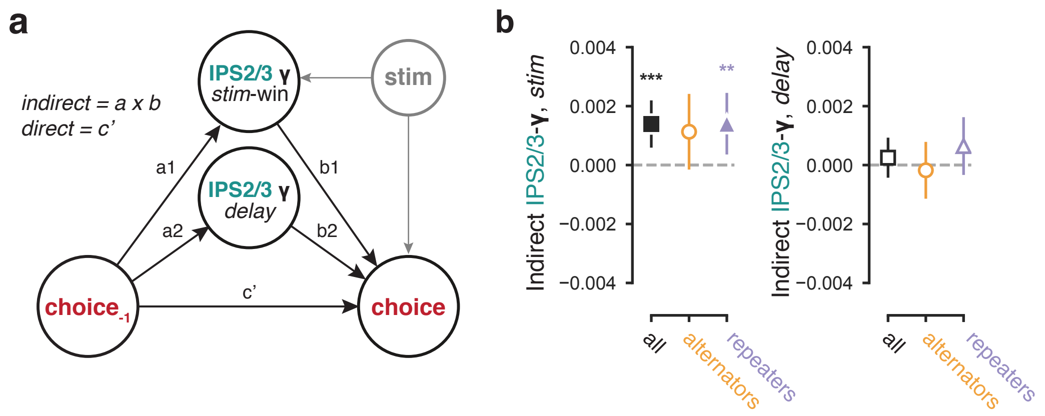

Figure S9. Mediation model controlling for effect of reference encoding in persistent activity during delay interval**.** ***(a)*** *Schematic of mediation model. All effects pointing to the binary choice were computed using logistic regression. In addition to the IPS2/3 gamma-band activity during the (test) stimulus interval, we included a proxy of the single-trial delay activity in IPS2/3 gamma, following test stimulus viewing. This proxy was decorrelated from the slowly varying choice history effects (Figure 5a) through baselining the delay activity with the single-trial baseline power. The result should have captured additional persistent activity induced by the preceding reference stimulus.* ***(b)*** *Indirect path estimates for the complete group (n = 60) as well as both subgroups (n = 25 alternators in orange, n = 34 repeaters in purple). Left: indirect path of IPS2/3 gamma-band during stimulus (as in Figure 6). All: t(59) = 4.145, p = 0.0001; alternators: t(24) = 1.879, p = 0.0724; repeaters: t(33) = 3.666, p = 0.0009. Right: indirect path of IPS2/3 gamma-band during delay (corrected with a single-trial baseline taking during the reference). All: t(59) = 0.677, p = 0.5008; t(24) = -0.424, p = 0.6755; repeaters: t(33) = 1.247, p = 0.2212. Data are shown as mean +- 95% confidence intervals, statistics from a simple t-test against zero or between groups. * 0.01 < p < 0.; *** p < 0.001; filled markers, p < 0.05.*

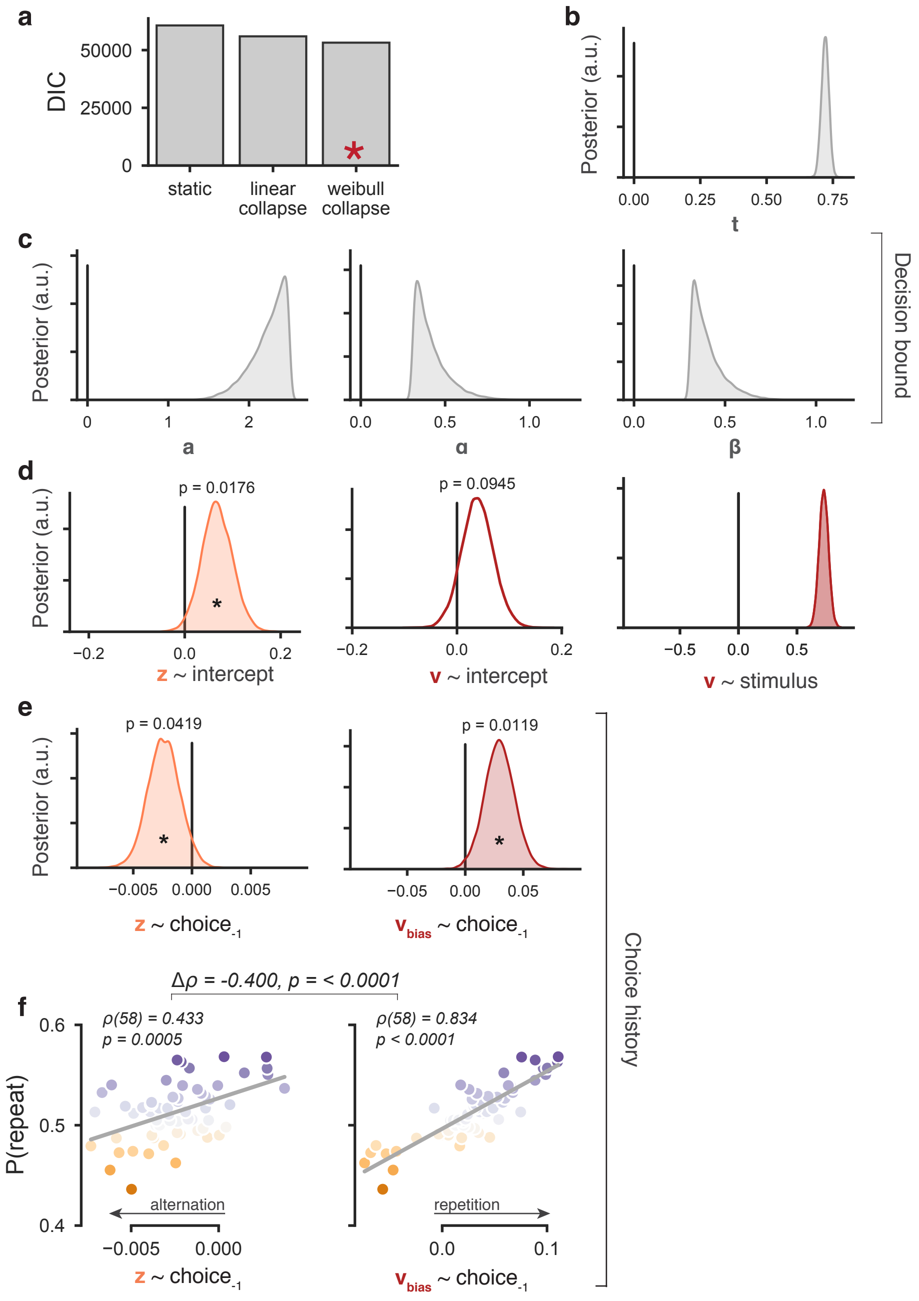

Figure S10. Full DDM model. *(**a**) Model comparison using the Deviance Information Criterion, between models with a static, linearly collapsing or nonlinearly collapsing bound (Fengler et al., 2021). In all three model variants, starting point and drift had an intercept and a dependence on previous responses, and drift additionally depended on the stimulus (Urai et al., 2019). The nonlinearly collapsing bound (described by a Weibull function, see Methods) best describes the data, as indicated by the lowest DIC. (**b-e**) Group-level posterior distributions of all parameters from the winning model. (**f**) Individual repetition behavior was better described by history-dependent shifts in drift rate than history-dependent shifts in starting point. Single correlation coefficients were computed using Spearman's rho, and the difference between the two using Steiger's test.*

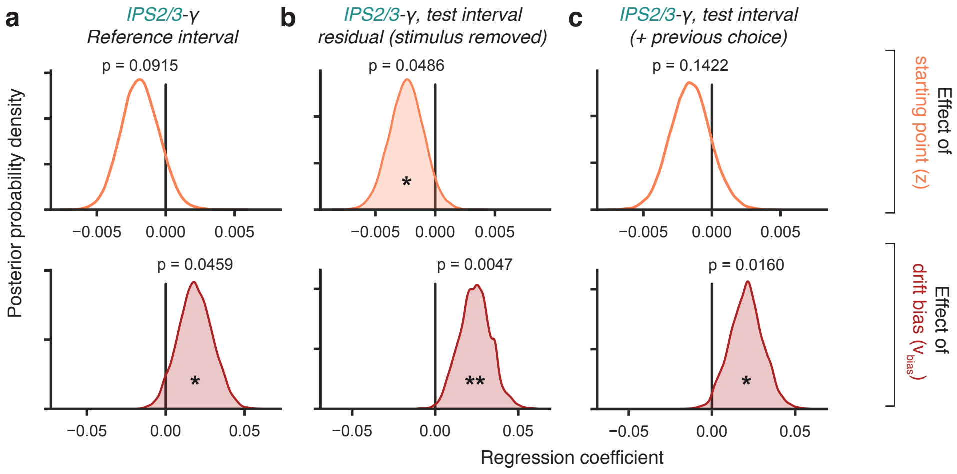

Figure S11. Control models for IPS2/3 gamma. *As Figure 7b, but (**a**) taking the parietal signal during the reference interval, (**b**) isolating stimulus-unrelated, intrinsic trial-to-fluctuations in IPS activity (including choice history signals), by removing the stimulus response in IPS2/3 gamma through linear regression, and (c) from a model that also includes a predictor for the previous choice.*

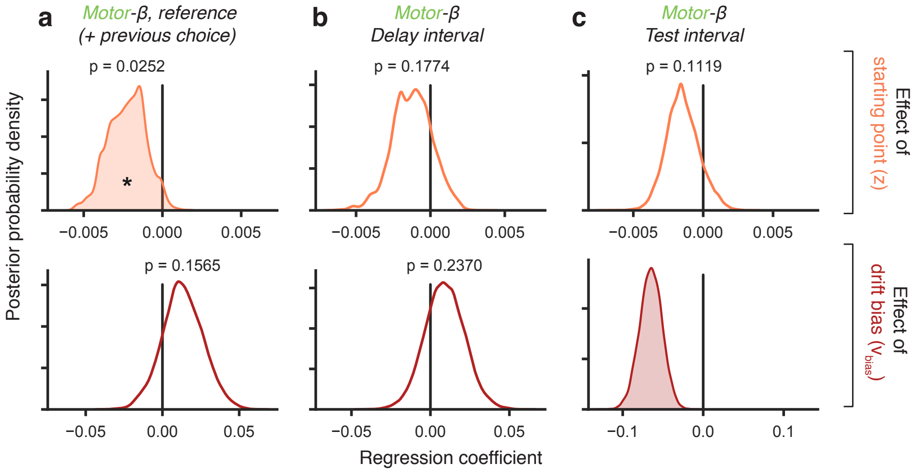

Figure S12. Control models for motor beta. *As Figure 7d, but (**a**) from a model that includes a predictor for the previous choice, (**b**) taking the motor beta signal during the pre-stimulus delay interval, and (**c**) taking the motor beta signal during the test stimulus interval. Note that the strongly negative effect on drift rate in (**c**) reflects preparation for the upcoming choice; during stimulus viewing, suppression of beta-band lateralization ramps up until the button press and tracks the upcoming motor response (Figure 3a).*

Under the assumption that motor beta tracks the build-up of the decision variable [4], one would expect the slope of the motor beta signal to be the primary predictor of drift. The current analysis shows that also the amplitude *of the motor beta signal during the decision interval strongly predicts drift. In fixed duration tasks like ours, the amplitude of a neural decision signal integrated across the decision interval is positively related to the slope of this neural signal, explaining the observed effect.*

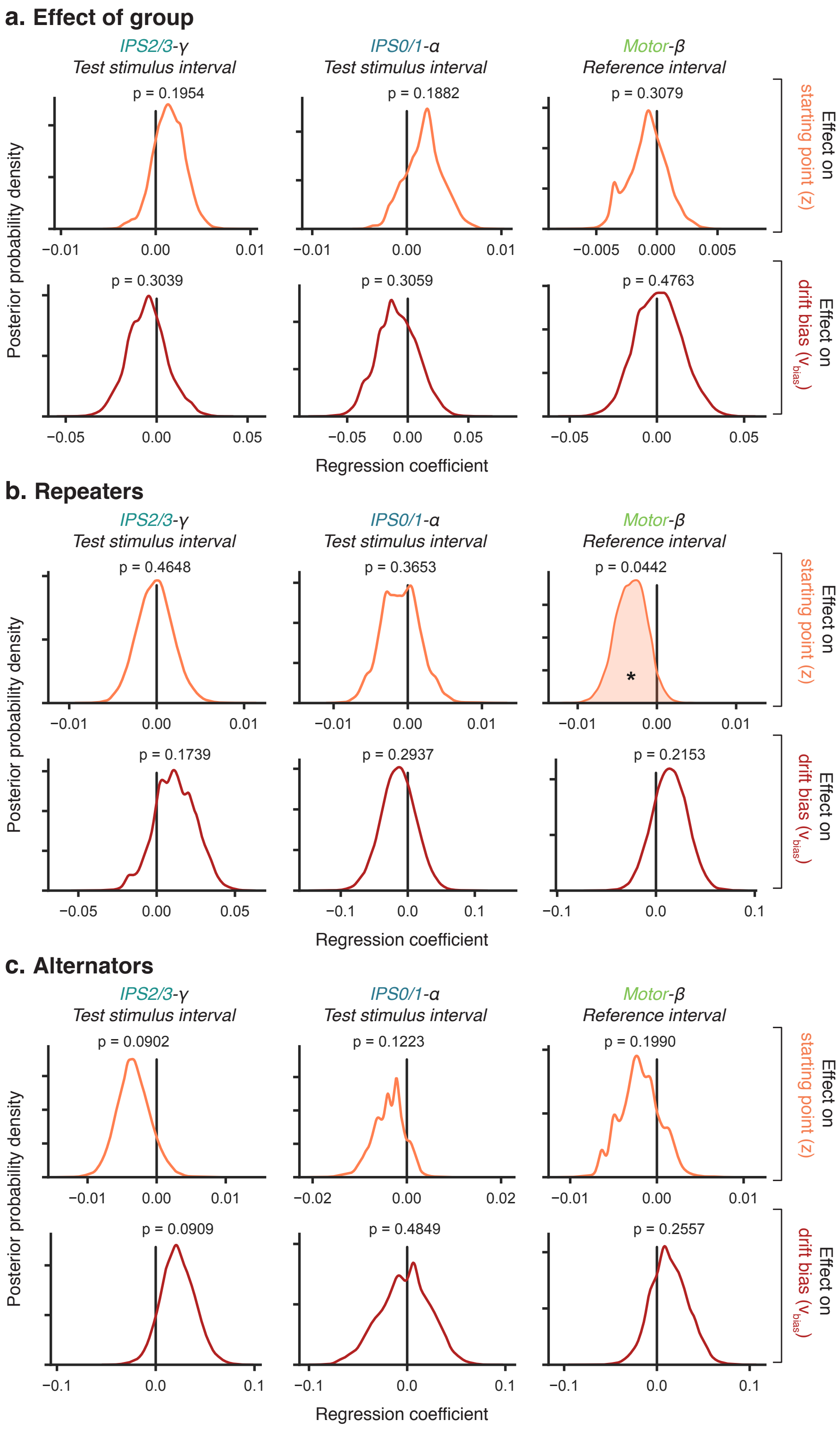

***Figure S13. HDDMnn models with subgroup interaction.** (**a**) Posterior probability density of the interaction of each neural regressor with subgroup. (**b**) As Figure 7, but for repeaters only. (**c**) As Figure 7, but for alternators only.*

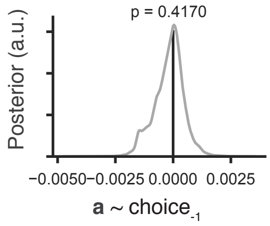

Figure S14. Control model with previous choices predicting bound height. *As Figure S9e, but with previous choices affecting trial-by-trial bound height.*

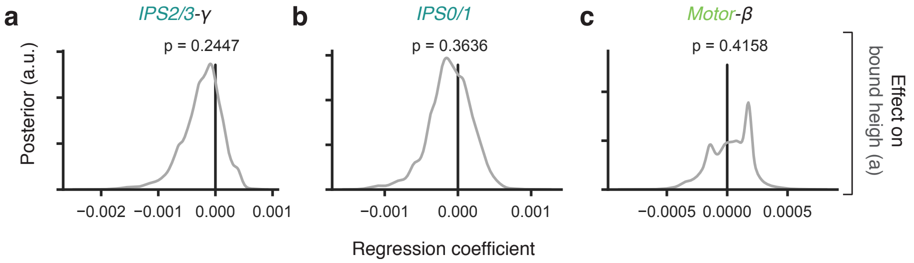

Figure S15. Control model with bound height. *As Figure 7d, but with each neural ignature loading onto trial-by-trial changes in the bound height.* ***(a)*** *IPS2/3 gamma,* ***(b)*** *IPS0/1 alpha,* ***(c)*** *motor (pooled M1, PMd/v, IPS/PCeS) beta.*

Supplementary References

1. Fründ, I., Wichmann, F. A. & Macke, J. H. Quantifying the effect of intertrial dependence on perceptual decisions. *J. Vis.* **14**, 9–9 (2014).
2. Hermoso-Mendizabal, A. *et al.* Response outcomes gate the impact of expectations on perceptual decisions. *Nat. Commun.* **11**, 1–13 (2020).
3. van Driel, J., Ridderinkhof, K. R. & Cohen, M. X. Not All Errors Are Alike: Theta and Alpha EEG Dynamics Relate to Differences in Error-Processing Dynamics. *J. Neurosci.* **32**, 16795–16806 (2012).
4. Murphy, P. R., Wilming, N., Hernandez-Bocanegra, D. C., Prat-Ortega, G. & Donner, T. H. Adaptive circuit dynamics across human cortex during evidence accumulation in changing environments. *Nat. Neurosci.* **24**, 987–997 (2021).
