# Supplementary material for: Persistent activity in human parietal cortex mediates perceptual choice repetition bias
Source: Nat Commun. 2022 Oct 12;13:6015. doi: 10.1038/s41467-022-33237-5 (PMC9556658; doi:10.1038/s41467-022-33237-5)
Supplement: Supplementary file 3 — Reporting Summary [file 41467_2022_33237_MOESM3_ESM.pdf]

nature portfolio

Corresponding author(s): Urai & Donner

Last updated by author(s): Aug 2, 2022

Reporting Summary

Nature Portfolio wishes to improve the reproducibility of the work that we publish. This form provides structure for consistency and transparency in reporting. For further information on Nature Portfolio policies, see our Editorial Policies and the Editorial Policy Checklist.

Statistics

For all statistical analyses, confirm that the following items are present in the figure legend, table legend, main text, or Methods section.

n/a	Confirmed	
☐	☒	The exact sample size (*n*) for each experimental group/condition, given as a discrete number and unit of measurement
☐	☒	A statement on whether measurements were taken from distinct samples or whether the same sample was measured repeatedly
☐	☒	The statistical test(s) used AND whether they are one- or two-sided<br>*Only common tests should be described solely by name; describe more complex techniques in the Methods section.*
☐	☒	A description of all covariates tested
☐	☒	A description of any assumptions or corrections, such as tests of normality and adjustment for multiple comparisons
☐	☒	A full description of the statistical parameters including central tendency (e.g. means) or other basic estimates (e.g. regression coefficient) AND variation (e.g. standard deviation) or associated estimates of uncertainty (e.g. confidence intervals)
☐	☒	For null hypothesis testing, the test statistic (e.g. *F*, *t*, *r*) with confidence intervals, effect sizes, degrees of freedom and *P* value noted<br>*Give P values as exact values whenever suitable.*
☐	☒	For Bayesian analysis, information on the choice of priors and Markov chain Monte Carlo settings
☐	☒	For hierarchical and complex designs, identification of the appropriate level for tests and full reporting of outcomes
☒	☐	Estimates of effect sizes (e.g. Cohen's *d*, Pearson's *r*), indicating how they were calculated

Our web collection on statistics for biologists contains articles on many of the points above.

Software and code

Policy information about availability of computer code

Data collection	MEG, eye-tracking and pupillometry data were collected using the acquisition software developed by the system manufacturer (MEG: CTF Systems Inc., version 5.4.2; Eye-tracking/pupil: SR Research). Behavioral data were collected using Matlab, using stimulus presentation functions from Psychtoolbox 3.<br><br>All code used to run the task, process data and generate figures is available at https://github.com/anne-urai/2022_Urai_choicehistory_MEG, as described in the Code availability statement.
Data analysis	Custom Matlab, Python and R code was used in combination with FieldTrip (MEG processing), HDDM (DDM fits) and lavaan (mediation) toolboxes.<br><br>All code used to run the task, process data and generate figures is available at https://github.com/anne-urai/2022_Urai_choicehistory_MEG, as described in the code availability statement. The GitHub repository includes specific information about e.g. Python environments.<br>The GitHub repository is archived at Zenodo.

For manuscripts utilizing custom algorithms or software that are central to the research but not yet described in published literature, software must be made available to editors and reviewers. We strongly encourage code deposition in a community repository (e.g. GitHub). See the Nature Portfolio guidelines for submitting code & software for further information.

Data

Policy information about availability of data

All manuscripts must include a data availability statement. This statement should provide the following information, where applicable:

- Accession codes, unique identifiers, or web links for publicly available datasets
- A description of any restrictions on data availability
- For clinical datasets or third party data, please ensure that the statement adheres to our policy

The processed behavioral and ROI data generated in this study have been deposited in the OSF database under accession code https://osf.io/v3r52/. The raw MEG data are available under restricted access for (due to the consent form used at time of data collection), and are available upon request from AEU.

Human research participants

Policy information about studies involving human research participants and Sex and Gender in Research.

Reporting on sex and gender	64 participants (aged 19-35 years, 43 women and 21 men) participated in the study after screening for psychiatric, neurological or medical conditions. All participants had normal or corrected to normal vision, were non-smokers, and gave their informed consent before the start of the study.
Population characteristics	64 participants (aged 19-35 years, 43 women and 21 men) participated in the study after screening for psychiatric, neurological or medical conditions. All participants had normal or corrected to normal vision, were non-smokers, and gave their informed consent before the start of the study.
Recruitment	Participants were recruited through the UKE Institute of Neurophysiology mailing list, and flyers put up at the university. This resulted in a sample largely consisting of medical students, thereby not forming a representative sample from the general population. However, our study investigates low-level decision processes which we believe to be largely unaffected by this possible selection bias.
Ethics oversight	The experiment was approved by the ethical review board of the University Medical Center Hamburg-Eppendorf (reference PV4648).

Note that full information on the approval of the study protocol must also be provided in the manuscript.

Field-specific reporting

Please select the one below that is the best fit for your research. If you are not sure, read the appropriate sections before making your selection.

☐ Life sciences ☒ Behavioural & social sciences ☐ Ecological, evolutionary & environmental sciences

For a reference copy of the document with all sections, see nature.com/documents/nr-reporting-summary-flat.pdf

Behavioural & social sciences study design

All studies must disclose on these points even when the disclosure is negative.

Study description	Quantitative: participants performed a behavioral task while we recorded their brain activity using MEG.
Research sample	64 participants (aged 19-35 years, 43 women and 21 men) participated in the study after screening for psychiatric, neurological or medical conditions. All participants had normal or corrected to normal vision, were non-smokers, and gave their informed consent before the start of the study. This sample was not intended to be fully representative of the population at large.
Sampling strategy	No statistical methods were used to pre-determine sample size but our sample size is equal to or larger than those reported in previous publications for comparable designs (e.g. Siegel et al., 2008, Neuron; Donner et al., 2009, Curr. Biol.; Wilming et al., 2020, Nat. Commun.). Of particular note, each of the 60 subjects attended 2 MEG sessions and we collected a median of 772 analyzable trials per participant (range=293-972), which is unusually large relative to existing studies and facilitates meaningful data analysis at the level of single subjects.
Data collection	MEG (CTF 275), eye-tracking (EyeLink), behavior (decision-making task coded in Matlab). Apart from the first author and the participant, a second experimenter licensed to perform MEG experiments was always present. Experiments were blinded to the pharmacological manipulation and to the individual choice history bias of each individual (which could only be completed post-hoc).
Timing	Data were collected in June-July 2014 (n = 20) and June-August 2015 (n = 44).
Data exclusions	After rejecting trials with excessive recording artefacts (see below), we discarded one additional participant with fewer than 100

Data exclusions	trials per session remaining. In total, 60 participants were included in the analysis.
Non-participation	Three participants did not complete the experiment and were thus excluded from analyses.
Randomization	Participants were not allocated in groups for these experiments (the pharmacological groups were not analyzed, see Methods).

Reporting for specific materials, systems and methods

We require information from authors about some types of materials, experimental systems and methods used in many studies. Here, indicate whether each material, system or method listed is relevant to your study. If you are not sure if a list item applies to your research, read the appropriate section before selecting a response.

Materials & experimental systems

n/a	Involved in the study	
☒	☐	Antibodies
☒	☐	Eukaryotic cell lines
☒	☐	Palaeontology and archaeology
☒	☐	Animals and other organisms
☒	☐	Clinical data
☒	☐	Dual use research of concern

Methods

n/a	Involved in the study	
☒	☐	ChIP-seq
☒	☐	Flow cytometry
☒	☐	MRI-based neuroimaging

nature portfolio | reporting summary

March 2021
